# How is missing data handled in cluster randomized controlled trials? A review of trials published in the NIHR Journals Library 1997–2024

**DOI:** 10.1177/17407745251378117

**Published:** 2025-10-04

**Authors:** Siqi Wu, Richard M Jacques, Stephen J Walters

**Affiliations:** School of Medicine and Population Health, University of Sheffield, Sheffield, UK

**Keywords:** Cluster randomized controlled trials, missing data, multiple imputation, NIHR Journals Library

## Abstract

**Background:**

Cluster randomized controlled trials are increasingly used to evaluate the effectiveness of interventions in clinical and public health research. However, missing data in cluster randomized controlled trials can lead to biased results and reduce statistical power if not handled appropriately. This study aimed to review, describe and summarize how missing primary outcome data are handled in reports of publicly funded cluster randomized controlled trials.

**Methods:**

This study reviewed the handling of missing data in cluster randomized controlled trials published in the UK National Institute for Health and Care Research Journals Library from 1 January 1997 to 31 December 2024. Data extraction focused on trial design, missing data mechanisms, handling methods in primary analyses and sensitivity analyses.

**Results:**

Among the 110 identified cluster randomized controlled trials, 45% (50/110) did not report or take any action on missing data in either primary analysis or sensitivity analysis. In total, 75% (82/110) of the identified cluster randomized controlled trials did not impute missing values in their primary analysis. Advanced methods like multiple imputation were applied in only 15% (16/110) of primary analyses and 28% (31/110) of sensitivity analyses. On the contrary, the review highlighted that missing data handling methods have evolved over time, with an increasing adoption of multiple imputation since 2017. Overall, the reporting of how missing data is handled in cluster randomized controlled trials has improved in recent years, but there are still a large proportion of cluster randomized controlled trials lack of transparency in reporting missing data, where essential information such as the assumed missing mechanism could not be extracted from the reports.

**Conclusion:**

Despite progress in adopting multiple imputation, inconsistent reporting and reliance on simplistic methods (e.g. complete case analysis) undermine cluster randomized controlled trial credibility. Recommendations include stricter adherence to CONSORT guidelines, routine sensitivity analyses for different missing mechanisms and enhanced training in advanced imputation techniques. This review provides updated insights into how missing data are handled in cluster randomized controlled trials and highlight the urgency for methodological transparency to ensure robust evidence generation in clustered trial designs.

## Introduction

Randomized controlled trials (RCTs) are considered the gold standard for evaluating the effectiveness of interventions in modern clinical trials,^1^ and the treatment effects are analysed at the individual level. However, when patients are recruited and randomly assigned in groups, for example, hospitals or schools, individuals within the same group may have similar characteristics such as age, race or medical history, as well as the response to treatment effects. In cluster randomized controlled trials (cRCTs), groups described above are defined as clusters, and the similarities within each group can be quantified by the intracluster correlation coefficient (ICC). Failure to account for clustering in the analysis stage can lead to underestimated standard errors, narrower confidence intervals, smaller *p* values and an increased likelihood of false-positive results, which can significantly overstate the effectiveness of the intervention effects.^2–5^

### Missing data in cRCTs

In cRCTs, missing data can occur in both the baseline and follow-up measurements. Apart from individual-level dropping out, clinical data may be missing at cluster level, that is the whole cluster is missing. The cluster level missingness can sometimes cause a high proportion of missing values.^6^ Missing values in practice are categorized into three types, based on whether the probability of recording a missing value is related to the observed data or missing data: Missing completely at random, missing at random (MAR) and missing not at random (MNAR).^7^

There are several handling methods for missing data in cRCTs. Common methods include complete case analysis (CCA),^8^ last observation carried forward (LOCF, especially for cRCTs with longitudinal measurements),^9^ cluster mean imputation (for continuous outcomes),^10,11^ Bernoulli single-draw-based strategy (for binary outcomes),^12,13^ predictive mean matching (PMM, for continuous outcomes)^14^ and regression imputation with fixed effects or random effects (suitable for both continuous and binary outcomes).^10^ These mechanisms are often combined with multiple imputation (MI) which can quantify the uncertainty of the imputed missing value.^15^

This study aimed to review, describe and summarize how missing primary outcome data are handled in reports of publicly funded cluster RCTs published in the UK National Institute for Health and Care Research (NIHR) Journals Library.

## Methods

This study reviewed how missing data were handled in cRCT reports published in the (NIHR https://www.journalslibrary.nihr.ac.uk/) Journals Library. The NIHR is a British government funder of health-related clinical researches which consists of five journals: Programme Grants for Applied Research (PGfAR), Public Health Research (PHR), Efficacy and Mechanism Evaluation (EME), Health and Social Care Delivery Research (HS&DR) and Health Technology Assessment (HTA). First, all published articles in the PGfAR, PHR, EME, HS&DR and HTA journals from 1 January 1997 to 31 December 2024 were manually archived, as the first NIHR Journals Library report was published in the HTA Journal in January 1997. Then, the first screening was processed on titles and abstracts of the NIHR papers to identify whether an RCT was reported. The second screening was based on the full text of the identified papers reporting RCTs to identify clinical reports with regard to cRCTs.

### Inclusion and exclusion criteria

For the first screening, papers that satisfied all the following including criteria were identified:

1. At least one (including pilot and feasibility) RCT was reported.
2. There was a chapter in the paper reporting the trial design and results.

The reason for the second criterion was to ensure the eligibility of the data extraction stage.

In the second screening, a report was excluded if it followed any of the excluding criteria:

1. Papers that did not include cRCTs.
2. Pilot or feasibility cRCT reports.

We were not interested in pilot or feasibility cRCTs, as missing data were often not considered a problem due to the objectives of these types of trials. All types of cRCT designs were included: parallel cohort, crossover, factorial and stepped-wedge.

### Data extraction

We extracted data from the identified cRCT papers on trial characteristics, including the trial design (parallel, crossover, factorial, stepped-wedge), follow-up type (closed cohort, open cohort, cross-sectional), number of arms, research hypothesis (superiority, non-inferiority, equivalence) and type of primary outcome (binary, count, time-to-event). The number of randomized clusters was defined as the number of clusters enrolled and randomized at the baseline. The number of completed clusters referred to the number of clusters that provided any outcome measurements for the primary analysis. Similarly, the number of individuals at baseline was defined as the total number of participants enrolled within the randomized clusters at baseline, while the number of individuals at primary analysis referred to the number of participants included in the primary analysis. The average cluster size was calculated by each trial’s number of individuals at baseline divided by the corresponding randomized clusters. Note that for non-parallel design trials, such as crossover and stepped-wedge designs, the calculation of average cluster size was not adjusted, which resulted in the average total number of participants in each cluster in the first period for these trials. The missing sample size was derived from the number of individuals at baseline minus the number of individuals at primary analysis, and the missing clusters were defined as the difference between randomized clusters and completed clusters. Missing individual proportion and missing cluster proportion were the proportion of the missing sample size and the number of clusters separately. ICCs were extracted for both the sample size calculation and the primary analysis. The observed effect size, calculated using the method proposed by Rothwell et al,^16^ was noted where available, and reasons for the absence of effect size calculations were recorded. We also evaluated the missing data handling methods applied in the primary analysis and identified the assumed missing data mechanism (MAR, MCAR or MNAR). Where sensitivity analyses were performed, the imputation models used were collected.

For cRCTs that employed MIs to handle missing data, additional details were collected, including the statistical model used for MI, the variables included in the imputation model, whether the imputation model accounted for clustering, and the number of iterations performed. In cases where a paper described multiple cRCTs, data for each trial were extracted separately under the same study title.

### Data processing and analysis

The extracted data were systematically compiled and imported into R (https://www.R-project.org/) for analysis. Descriptive statistics were calculated to summarize sample sizes, missing data proportions and the frequency of missing data handling methods. Trends in the adoption of imputation techniques and other handling methods were analysed over time.

## Results

**Figure 1.**
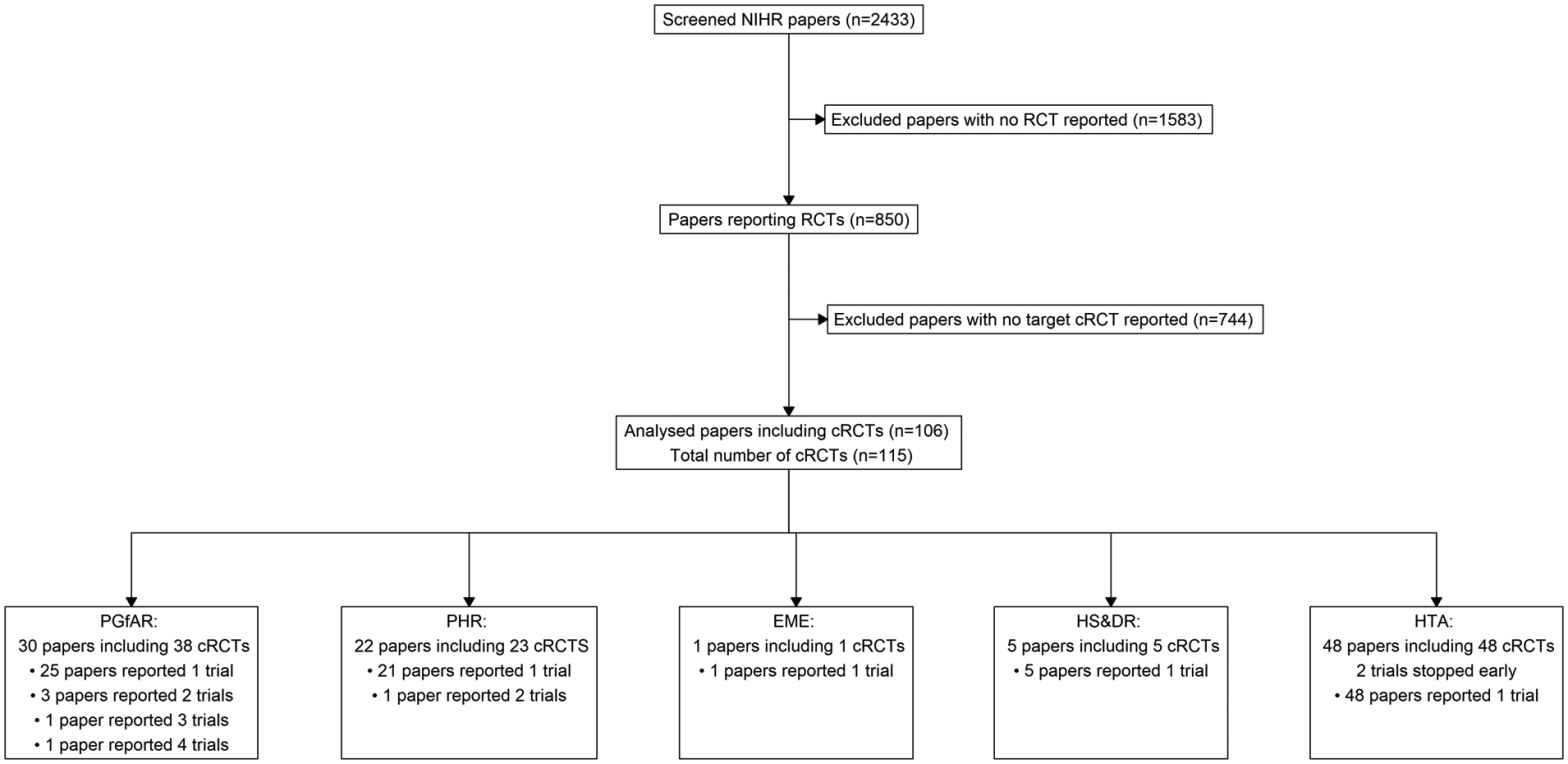
Flow diagram of the screening process for the sample of 110 cluster randomized trials included in this review.

The screening process is shown in Figure 1. In total, 2433 papers were published in the NIHR database between 1 January 1997 and 31 December 2024. Following screening of titles and abstracts, 850 papers were identified as reporting RCTs. After applying the exclusion criteria, 744 papers were excluded that did not report a cRCT or reported feasibility/pilot cRCTs. In total, 106 eligible papers reporting the results of 115 cRCTs were analysed. Two papers reported the results of trials that stopped early.^17,18^

Table 1 provides a summary of the numerical variables extracted from the reviewed cRCTs. The number of individuals at baseline across trials showed a large variability, ranging from as few as 91 participants to as many as 626,625, with a median of 1309 participants. This variability reflects the diverse nature of cRCTs included in the review, spanning different research areas and intervention scales. The median value is more representative than the mean (18,618) due to the presence of outliers, such as some large public health studies. Similarly, the number of individuals at primary analysis also varied widely, with a median of 916 participants.

**Table 1.** Summary table of the numeric variables in the NIHR review.

	Mean	Standard deviation	Min	25th quantile	Median	75th quantile	Max
**Basic characteristics**							
Number of individuals at baseline (n = 109)	18,618	72,618	97	581	1309	3260	626,625
Number of individuals at primary analysis (n = 109)	13,820	62,243	89	409	916	2383	582,675
Randomized clusters (n = 113)	84	172	6	26	45	78	1523
Completed clusters (n = 108)	81	162	6	25	44	76	1382
Average cluster size (n = 108)	443	2191	1	13	29	80	20,918
**Missing data summary**							
Missing sample size (n = 109)	4799	18,705	−18	67	252	837	125,602
Missing clusters (n = 108)^a^	4	16	0	0	0	1	141
Missing individual proportion (%) (n = 109)	25	18	−5	12	24	35	81
Missing cluster proportion (%) (n = 108)	2	5	0	0	0	2	29
**Summary of ICC and effect size**							
ICC for sample size calculation (n = 97)	0.06	0.07	0.00	0.02	0.05	0.06	0.50
ICC for primary analysis (n = 58)	0.06	0.09	−0.02	0.00	0.02	0.09	0.59
Standardized effect size (n = 89)	0.11	0.14	0.00	0.03	0.07	0.12	1.03

a No cluster-level missingness was reported in crossover and stepped-wedge design trials.

For the missing data information, the minimum missing sample size was −18^19^ because some of the trials were open cohorts which resulted in larger sample sizes at baseline than sample sizes at primary analysis. The maximum missing sample size was 125,602^20^ which was much larger in scale, compared with the median (252) and the mean (4799). The proportion of missing data at the individual level averaged 25%, with a median of 24%. This suggests that, on average, about one-quarter of participants were not included in the primary analysis. Furthermore, the range of missing proportions (from −5% to 81%) highlights disparities in trial execution. Note that negative values in some open cohort studies occurred when the completed sample size exceeded the randomized size due to additional participants recruited during follow-up phases (open cohort).

At the cluster level, the number of randomized clusters ranged from 6 to 1523, with a median of 45 clusters. The number of completed clusters was slightly lower, with a median of 44, reflecting the occasional loss of entire clusters. Missing clusters were relatively uncommon, with 4% of missingness on average. Most trials having no missing clusters (median = 0) and only a small subset reporting substantial cluster losses (maximum = 141). Two trials did not record the number of randomized clusters due to either poor reporting of cRCT^21^ or the early stop.^18^ Seven cRCTs did not report the number of clusters analysed, including the previous two trials, one other trial that stopped early^17^ and four trials within the same paper that did not have a systematic guideline of reporting in early years.^20^

The ICC, a key parameter in cRCTs, was only reported in 58 out of 110 trials. In general, the observed ICC showed relatively small values in both sample size calculations (median = 0.05) and primary analyses (median = 0.02). The largest ICC observed was 0.59, emphasizing the potential for high clustering effects in certain trials. These findings align with the literature suggesting that clustering effects are typically small but can vary significantly depending on the trial context.^5^ The observed standardized effect size, calculated using the method proposed by Rothwell et al.,^16^ was consistently small, with a median of 0.07 and a mean of 0.11. These small effect sizes indicated the challenges in detecting treatment effects in cRCTs, particularly when combined with high levels of missing data or weak intervention effects.

The summary of trial designs of the identified 115 trials is presented in Table 2. The majority of cRCTs (89%) applied a parallel design, reflecting its simplicity and widespread applicability in clinical research. Factorial designs (5%), crossover designs (2%) and stepped-wedge designs (4%) were much less common. For follow-up types, closed cohorts dominated (83%), as they provide a fixed and stable population for analysis. Cross-sectional follow-ups (11%) were also notable, primarily in studies where different participants were recruited at each time point. Open cohorts (6%) were rare, likely because their dynamic nature complicates data analysis and missing data management. New participants were recruited in the follow-up phase for each cluster, and different participants provided clinical measurements at check points. Continuous outcomes were the most frequently reported primary outcome type (62%), followed by binary outcomes (30%). Count data (6%) and time-to-event outcomes (2%) were relatively uncommon.

**Table 2.** The frequency of the basic trial characteristics of the identified NIHR cRCTs.

Category variables	N (%)
**Trial design (n** **=** **115)**	
Parallel	102 (89)
Factorial	6 (5)
Crossover	2 (2)
Stepped-wedge	5 (4)
**Type of follow-up (n** **=** **115)**	
Closed cohort	95 (83)
Open cohort	7 (6)
Cross-sectional	13 (11)
**Primary outcome data type (n** **=** **115)**	
Continuous	71 (62)
Binary	35 (30)
Count number	7 (6)
Time to event	2 (2)
**Missing mechanism (n** **=** **115)**	
MAR	26 (22)
MCAR	2 (2)
MNAR	1 (1)
Not reported	86 (75)
**Missing data handling method in primary analysis (n** **=** **115)**	
Not reported	53 (45)
CCA	33 (29)
LOCF	2 (2)
Mean imputation	6 (5)
MI	16 (14)
Regression imputation	3 (3)
Missing indicator	2 (2)
**Sensitivity analysis imputation model (n** **=** **115)**	
Not reported	78 (67)
LOCF	2 (2)
MI	31 (27)
Regression imputation	1 (1)
LOCF, mean imputation	1 (1)
LOCF, MI	1 (1)
LOCF, regression imputation	1 (1)
**Imputation applied in either primary analysis or sensitivity analysis (n** **=** **115)**	
Yes	61 (53)
No (including not reported)	54 (47)
**Combination of handling methods in primary and sensitivity analysis (Primary – Sensitivity) (n** **=** **61)**	
CCA **–** MI	16 (26)
MI **–** Not reported	14 (23)
Not reported **–** MI	10 (16)
Mean imputation **–** Not reported	4 (6)
Regression imputation **–** Not reported	3 (5)
LOCF **–** Not reported	2 (3)
MI **–** MI	2 (3)
Mean imputation **–** MI	2 (3)
CCA **–** LOCF	1 (2)
CCA **–** LOCF, mean imputation	1 (2)
CCA **–** LOCF, MI	1 (2)
CCA **–** LOCF, regression imputation	1 (2)
CCA **–** Regression imputation	1 (2)
Missing indicator **–** MI	1 (2)
Missing indicator **–** Not reported	1 (2)
Not reported **–** LOCF	1 (2)

The reporting of missing mechanism is an essential information to justify the missing data handling methods. Overall, the assumed missing mechanism could not be determined from 75% (86) of the identified trials. MAR was reported in 26 (22%) trials, while MCAR and MNAR were each reported in just 2 and 1 trial. Nearly half of the trials (45%) did not report any specific method for handling missing data in the primary analysis, while CCA was the most commonly reported method applied in 33 trials (29%). MI, the gold standard under MAR, was used in only 9 out of 26 trials that reported MAR assumption, while 11 trials under MAR assumption ignored the missing data (CCA).

In total, MI was used in the primary analysis for 16 trials, and the details of these 16 MIs were summarized in Table 3. Most of the trials employed a parallel design and closed cohort follow-up, which was consistent with the dominance of this design in cRCTs overall. Continuous outcomes were the most frequently analysed using MI, aligning with the statistical ease of applying MI to continuous data under the MAR assumption. Binary outcomes also featured prominently, often requiring logistic regression-based imputation models. Count and time-to-event outcomes were less frequently addressed. All reported missing mechanisms were MAR, but nearly half of the trials (7/16) did not record the missing mechanism. Many trials applied PMM or regression-based imputation methods, both of which are standard approaches under MAR. The number of imputed data sets varied across trials, with some reporting as many as 100 or more. Overall, the details of MI were not recorded in sufficient detail to determine whether the MI was appropriate, such as missing data mechanisms, MI models and included variables.

**Table 3.** Summary of the MI models applied in primary analysis of the identified cRCTs.

Study	Trial design	Follow-up type	Primary outcome type	Missing mechanism	MI model	Variables included	Imputation considering cluster?	Number of imputed data sets
Killaspy et al.^22^	Parallel	Closed cohort	Continuous	MAR	NA	Life skill profile score, length of illness	NA	NA
Moniz-Cook et al.^23^	Parallel	Open cohort	Continuous	MAR	Random effect model	Baseline characteristics, cluster variable	Yes	NA
Thompson et al.^24^	Parallel	Closed cohort	Continuous	NA	NA	Baseline covariates	No	5
Thompson et al.^24^	Parallel	Closed cohort	Continuous	NA	NA	Baseline covariates	NA	NA
Thompson et al.^24^	Parallel	Closed cohort	Continuous	NA	NA	Baseline covariates	NA	NA
Ballard et al.^25^	Parallel	Closed cohort	Continuous	NA	NA	NA	NA	NA
Price et al.^26^	Parallel	Closed cohort	Binary	NA	Predictive mean matching	NA	NA	NA
Foy et al.^27^	Factorial	Cross-sectional	Binary	MAR	NA	Full imputation model	NA	100
Foy et al.^27^	Factorial	Cross-sectional	Binary	MAR	NA	Full imputation model	NA	100
Humphrey et al.^28^	Parallel	Closed cohort	Continuous	MAR	REALCOM impute	Constant trial group, partially observed outcome scores and a range of demographic data	Yes	1000
Salisbury et al.^29^	Parallel	Closed cohort	Continuous	NA	Predictive mean matching	Baseline variables, cluster variables and aggregate cost variables	Yes	40
Mouncey et al.^30^	Parallel	Cross-sectional	Continuous	MAR	Predictive mean matching	Site level and patient level covariates	Yes	50
Heller et al.^31^	Parallel	Closed cohort	Continuous	NA	NA	Baseline characteristics, cluster variable	Yes	50
Ring et al.^32^	Parallel	Closed cohort	Continuous	MAR	Predictive mean matching	Not reported	NA	20
Estcourt et al.^33^	Crossover	Closed cohort	Binary	MAR	NA	NA	NA	NA
Wright et al.^34^	Parallel	Closed cohort	Continuous	MAR	Random effect model	Baseline covariates	Yes	NA

a NAs in the table refer to information that was not reported or could not be extracted from the cRCT reports.

Mean imputation (5%), regression imputation (3%) and LOCF (2%) were seldom applied in the primary analysis phase. The underreporting of missing data mechanisms and handling methods limits the transparency and interpretability of trial findings and highlights a huge gap in the application of reporting standards, such as the CONSORT extension for cluster trials.

For the sensitivity analysis part, only 33% (37/115) of trials reported performing sensitivity analyses to assess the robustness of their findings under alternative missing data assumptions. Among these, MI was the most commonly applied method (27%), reflecting its growing recognition as a robust approach for handling missing data. Other methods, such as CCA and LOCF, were rarely used in sensitivity analyses. The lack of sensitivity analyses in most trials limits the ability to evaluate the impact of missingness assumptions on study conclusions, particularly for MNAR scenarios.

**Figure 2.**
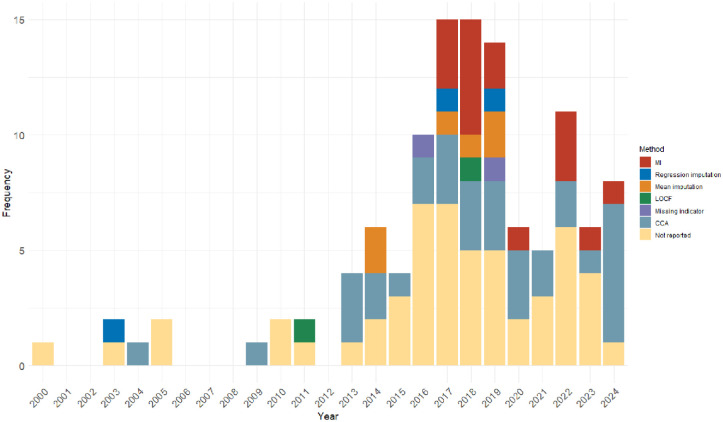
Distribution of missing data handling methods applied in primary analysis over years.

Among the 27 years from 1997 to 2024, eligible cRCTs were identified for 19 years. The distribution of missing data handling methods being applied to these cRCTs in the primary analysis is shown in Figure 2. The first identified cRCT was published in November 2000, and the first missing data imputation method was reported in 2003. MI, the golden rule for missing data imputation, was first applied in 2017 and has been widely used since then. The mean imputation method was also often used after 2014. However, the majority of the identified cRCTs either ignored the missing data (CCA) or did not report them.

To show a clearer trend of whether the imputation methods were overall applied in the cRCTs, the missing data handling methods were categorized into two classes: No imputation applied (CCA and not reported) and imputation applied (all other methods). In total, imputation methods were applied in more than half (53%) of the identified cRCTs in either primary analysis or sensitivity analysis. In total, 54 cRCTs did not handle missing data at all. Among the 61 cRCTs that took action of missing data, the combination of CCA in primary analyses and MI in sensitivity analyses was the most common (16), following with MI in primary analyses and no imputation applied in sensitivity analyses (14).

**Figure 3.**
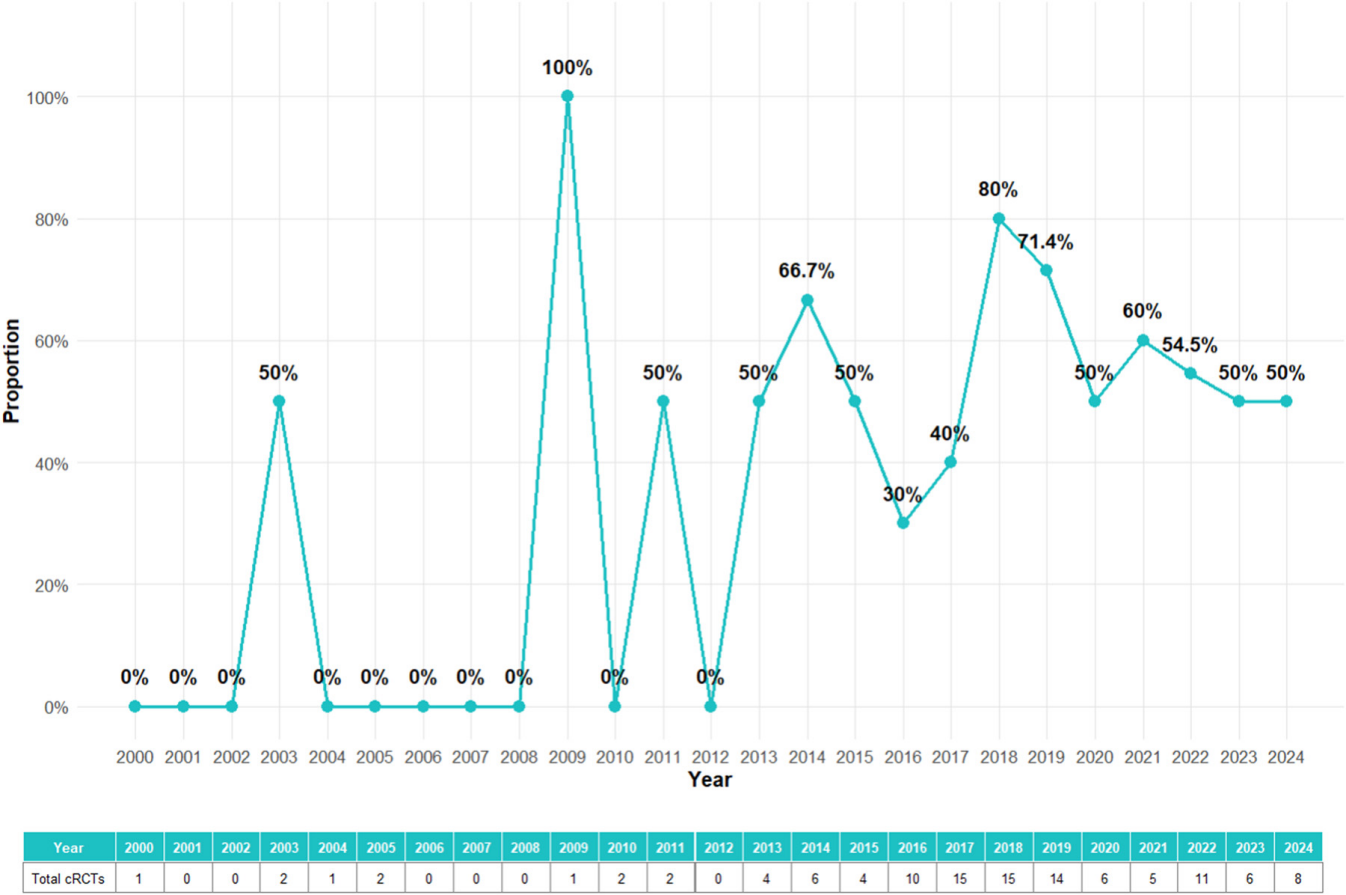
Trend of imputation applied in either primary analysis or sensitivity analysis over years.

The trend of how missing data handled over time is presented in Figure 3. During the early period (2000–2010), there was limited reporting of missing data handling methods in cRCTs. Imputation methods were almost entirely absent, with most trials either relying on CCA or not addressing missing data explicitly. This highlights a lack of awareness and methodological guidance for handling missing data in earlier years. From 2011 to 2016, there was an increase in the use of imputation methods began around 2011, likely reflecting the growing availability of advanced statistical tools and increased awareness of their importance in handling missing data. However, the majority of trials during this period still either ignored missing data or relied on simpler methods like CCA. In recent years (2017–2024), there was a sharp rise of the number of cRCTs that applied imputation methods in either primary analysis or sensitivity analysis. As an essential criteria to justify missing data handling methods, the assumed missing mechanism could only be extracted from 25% of the identified cRCT reports. The reporting rate of missing mechanism over time was shown in the supplemental materials. The rate increased after 2012, but was generally lower than 50% even in recent years. Overall, the increasing adoption of imputation methods, especially MI, highlights a growing commitment to addressing missing data appropriately in cRCTs. Despite these improvements, a significant proportion of trials still do not report missing data handling methods properly, even in recent years.

## Discussion

This literature review provides an updated evaluation of missing data handling practices in cRCTs published in the NIHR Journals Library between 1997 and 2024. Our findings demonstrate significant progress in the use of advanced methods such as MI over the last decade but also reveal persistent gaps in reporting and handling missing data in cRCTs.

### Comparison with previous reviews

We have identified two reviews that studied similar objectives. Díaz-Ordaz et al.^6^ reviewed 132 cRCTs published in 2011, and Fiero et al.^35^ reviewed 86 cRCTs published between August 2013 and July 2014. The results align with the findings of the two previous reviews, which highlighted the predominance of CCA and the poor reporting of missing data mechanisms. However, compared with these earlier reviews, our study observed an increase in the application of MI, particularly after 2017, as well as a broader application of parametric imputation methods such as linear and logistic mixed effects models. These changes reflect growing awareness and accessibility of statistical methods, as well as increased emphasis on handling missing data appropriately in methodological guidelines.

Despite these improvements, the proportion of trials that fail to report missing data mechanisms or handling methods remains concerning. In our review, 74% of trials did not specify the mechanism of missingness, and 45% did not report any method for handling missing data. These rates are similar to those reported a decade ago, indicating that underreporting remains a persistent problem in cRCTs. The lack of transparency and adherence to reporting guidelines, such as the CONSORT extension for cluster trials, undermine the interpretability and reproducibility of trial results.

### Trends in missing data handling

Our findings highlight an encouraging trend in recent years in the increased use of MI and other imputation methods in primary analyses. MI, recognized as the gold standard for handling missing data under the MAR assumption,^36^ was applied in 9 out of 26 cRCTs that assumed MAR, and overall applied in 14% of primary analyses and 28% of sensitivity analyses in the reviewed trials. However, its adoption remains far from universal, and the use of single imputation methods, such as LOCF, persists despite their well-documented limitations.^9^

Sensitivity analyses were reported in only 35% of trials, limiting the evaluation of robustness under alternative assumptions about missingness. This is particularly concerning given the potential for MNAR mechanisms in cRCTs, which require sensitivity analyses to assess the impact of departures from MAR assumptions. Furthermore, 45% of the identified cRCTs did not take any action on missing data in either primary or sensitivity analysis phase. Further restrictions on cRCT missing data reports should be considered despite the rising trend of imputation applications in this decade.

### Strengths and limitations of the review

This study benefits from its comprehensive scope, spanning over 25 years of cRCT publications in the NIHR Journals Library. By focusing on a single, well-defined database, we ensured consistency in study selection and data extraction. In addition, our review provides an updated perspective on missing data practices, addressing a key gap in the literature since the last reviews were conducted nearly a decade ago.

However, several limitations should be noted. First, the study is restricted to NIHR-funded trials, which may limit generalizability to cRCTs conducted in other settings or funded by other organizations. Second, our reliance on published reports may underestimate the true extent of missing data or the sophistication of handling methods, as reporting practices vary widely. Finally, our review did not evaluate the impact of missing data handling on trial outcomes, which would require additional analysis of primary data.

### Implications and recommendations

Our findings emphasize the need for greater transparency and restrictions in reporting and handling missing data in cRCTs. To improve practice, we recommend the following:

1. Adherence to reporting guidelines, such as the CONSORT extension for cluster trials, to ensure the accurate recording of missing data mechanisms and handling methods.
2. Broader adoption of advanced statistical methods, such as MI and parametric model-based approaches, which are valid under plausible assumptions and preserve statistical power.
3. Routine inclusion of sensitivity analyses to evaluate the robustness of findings under alternative missing data assumptions, particularly in the presence of MNAR mechanisms.
4. Continued development of accessible tools and training to support the implementation of advanced missing data methods in cRCTs.

## Conclusion

While progress has been made in handling missing data in cRCTs, significant gaps remain in reporting and the adoption of advanced methods. By providing an updated perspective, this review highlights the need for continued efforts to improve transparency, methodological credibility and the robustness of cRCT findings. Future research should focus on developing practical tools to facilitate the application of advanced imputation methods and on reporting their impact carefully with essential information.

## Supplemental Material

sj-png-1-ctj-10.1177_17407745251378117 – Supplemental material for How is missing data handled in cluster randomized controlled trials? A review of trials published in the NIHR Journals Library 1997–2024 Supplemental material, sj-png-1-ctj-10.1177_17407745251378117 for How is missing data handled in cluster randomized controlled trials? A review of trials published in the NIHR Journals Library 1997–2024 by Siqi Wu, Richard M Jacques and Stephen J Walters in Clinical Trials
