# Supplementary figures and images for: How is missing data handled in cluster randomized controlled trials? A review of trials published in the NIHR Journals Library 1997–2024
Source: Clin Trials. 2025 Oct 4;23(1):75–84. doi: 10.1177/17407745251378117 (PMC12909601; doi:10.1177/17407745251378117)

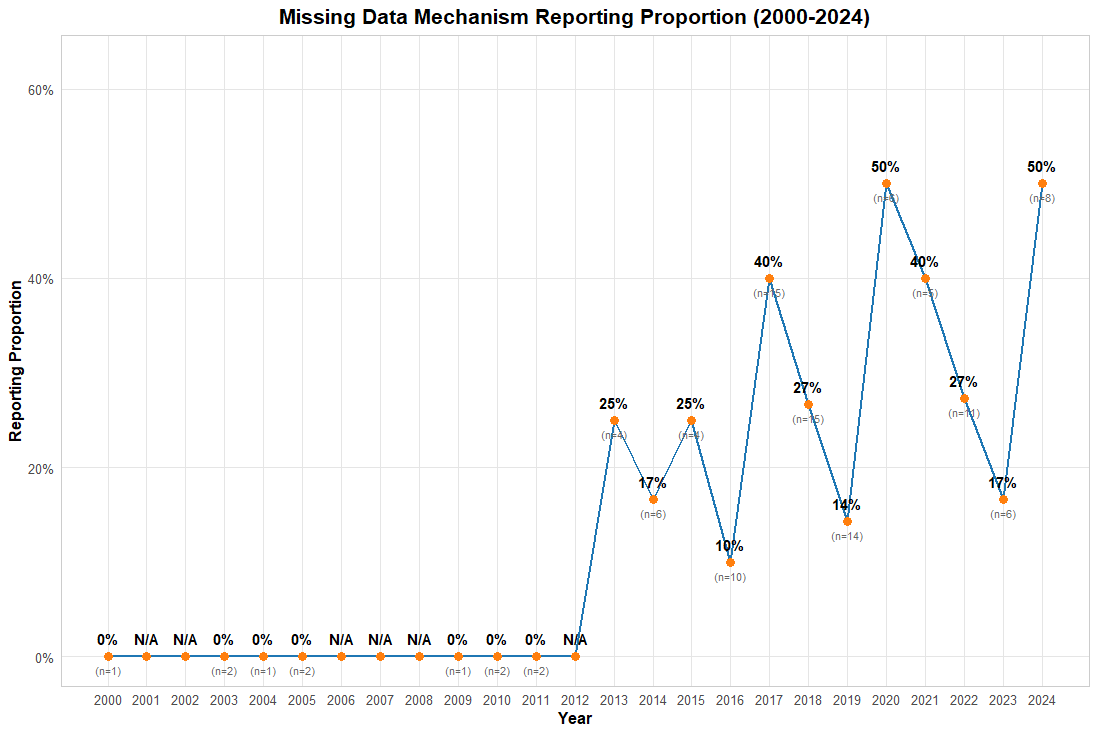

Supplement: sj-png-1-ctj-10.1177_17407745251378117 – Supplemental material for How is missing data handled in cluster randomized controlled trials? A review of trials published in the NIHR Journals Library 1997–2024 [file sj-png-1-ctj-10.1177_17407745251378117.png]
